# Microcomputed Tomography with Diffraction-Enhanced Imaging for Morphologic Characterization and Quantitative Evaluation of Microvessel of Hepatic Fibrosis in Rats

**DOI:** 10.1371/journal.pone.0078176

**Published:** 2013-10-21

**Authors:** Jinghao Duan, Chunhong Hu, Shuqian Luo, Xinyan Zhao, Tailing Wang

**Affiliations:** 1 College of Biomedical Engineering, Tianjin Medical University, Tianjin, China; 2 College of Biomedical Engineering, Capital Medical University, Beijing, China; 3 Liver Research Center, Beijing Friendship Hospital, Capital Medical University, Beijing, China; 4 Department of Pathology, China-Japan Friendship Hospital, Beijing, China; University of Pisa, Italy

## Abstract

**Backgroud:**

Hepatic fibrosis can lead to deformation of vessel morphology and structure. In the present feasibility study, high-resolution computed tomography (CT) using diffraction-enhanced imaging (DEI) was used to represent three-dimensional (3D) vessel microstructures of hepatic fibrosis in rats and to differentiate different stages of hepatic fibrosis using qualitative descriptions and quantitative measurement of microvessels.

**Material and Methods:**

Three typical specimens at different stages, i.e., mild, moderate and severe hepatic fibrosis, were imaged using DEI at 15 keV without contrast agents. The correspondence between DEI-CT images and histopathological findings was determined. The 3D visualizations from different stages of hepatic fibrosis were presented using DEI-CT. Additionally, Qualitative descriptions and quantitative evaluation of vessel features, such as vessel trend, vascular distortion deformation, thrombus formation and texture features on the inner wall of the vessel, were performed.

**Results:**

DEI-CT produced high-resolution images of the vessel microstructures in hepatic fibrosis that corresponded to information on actual structures observed from the histological sections. Combined with the 3D visualization technique, DEI-CT enabled the acquisition of an accurate description of the 3D vessel morphology from different stages of hepatic fibrosis. Qualitative descriptions and quantitative assessment of microvessels demonstrated clear differences between the different stages of hepatic fibrosis. The thrombus inside the vessel of severe liver fibrosis was accurately displayed, and corresponding analysis can provide an exact measurement of vessel stenosis rate.

**Conclusions:**

DEI-CT may allow morphologic descriptions and quantitative evaluation of vessel microstructures from different stages of hepatic fibrosis and can better characterize the various stages of fibrosis progression using high-resolution 3D vessel morphology.

## Introduction

Hepatic fibrosis refers to the excessive accumulation of extracellular matrix proteins, including collagen, that occurs in many chronic hepatic diseases, and it may result in hepatic cirrhosis and decreased liver functional reserve [1,2]. In general, hepatic fibrosis can cause vascular diseases of varying severity, and thus, it can lead to deformation of vessel morphology and structure. Conventionally, liver biopsy has remained the gold standard for the assessment of hepatic fibrosis. However, this technique has some inherent limitations, such as invasiveness and sampling error [2,3]. Vessel imaging plays a vital role in the diagnosis of hepatic fibrosis [4-6]. Moreover, imaging can assist in the early detection of hepatic fibrosis and the objective evaluation of the degree of fibrosis, which will be very helpful when studying the development of hepatic fibrosis [3,7]. However, conventional imaging techniques, such as conventional radiography, computed tomography (CT), ultrasonography, positron emission tomography (PET) and magnetic resonance imaging (MRI), have proved insensitive for detection of mild to moderate hepatic fibrosis [7]. As a novel imaging techniques, magnetic resonance elastography has been able to stage fibrosis or diagnose mild disease [7,8]. The existing imaging techniques can provide vessel images, but are unable to resolve the microvasculature due to spatial resolution and contrast limitations. Contrast agents are often used to highlight vessels for the observation of microvessels in these imaging modalities. However, contrast agents occasionally cause adverse reactions ranging from minor to severe, sometimes resulting in death [9].

Conventional radiography depends on differences in linear attenuation coefficients between soft tissues. Unfortunately, the attenuation coefficient variations between the vessels and the surrounding tissues are quite small and the undesired scattering blurs the image. Thus, imaging microvessels is difficult with conventional radiography. Herein x-ray phase-contrast imaging (PCI) is presented to overcome this limitation; PCI is an emerging imaging technique that provides high contrast and high resolution of biological soft tissues compared to conventional radiography [10-12]. For biological soft tissue, PCI has approximately 1000 times greater sensitivity than conventional radiography in the detection of minute density changes, and its spatial resolution can be on the order of microns or even sub-microns. Diffraction-enhanced imaging (DEI) is one of the most effective and practical PCI techniques and has been widely applied to the investigation of biological soft tissues [4,6,13-15]. In recent years, DEI has also been introduced into CT, and DEI-CT possesses excellent properties over conventional absorption-based CT when imaging soft tissues, such as breast [16-18], brain [19,20], and liver [5,21]. Especially in vessel imaging fields, DEI-CT as a nondestructive three-dimensional (3D) technique enables the visualization of the vascular system down to micrometer levels without contrast agents wherein it clearly displays 3D vessel microstructures [5,21]. High-resolution 3D visualization of the vascular network can improve the detection of vascular diseases hidden by superimposed structures that suffer from the planar radiography mode and can better portray the anatomical and pathological features of the vessels.

In this work, DEI was used to image different stages of hepatic fibrosis samples in rats, and 3D vessel microstructures of hepatic fibrosis were visualized using DEI-CT without contrast agents. The morphologic and quantitative assessments, which characterized anatomical properties and pathological features of microvessels, were performed. The purpose of this study was to investigate whether the high-resolution DEI-CT technique could be used to differentiate different stages of hepatic fibrosis using qualitative descriptions and quantitative measurement of microvessels.

## Materials and Methods

### Hepatic Fibrosis Specimens

All experiments and procedures involving animals were approved by the animal welfare committee of Capital Medical University. Experiments were performed with female Wistar rats (Charles River Laboratories; Beijing, China), each weighing 200 g ± 20 [standard deviation]. The hepatic fibrosis specimens from the rats, which were induced by human albumin, were prepared at the Liver Research Centre of Beijing Friendship Hospital at Capital Medical University. The detail of hepatic fibrosis model can be provided in the corresponding references 22,23. The rats were deeply anesthetized and euthanized before the collection of liver specimens. Subsequently, the livers were quickly removed and fixed in 10% buffered formalin prior to imaging. Three typical specimens on which to perform imaging experiments were chosen by a pathologist; the chosen specimens represented mild, moderate and severe hepatic fibrosis. Based on newly proposed fibrosis staging criterion [23], they corresponded to different stages: stage 1, stage 3 and stage 5. The specimens were cut into small pieces of 6 × 4 mm with a thickness of approximately 2-4 mm. After imaging, the specimens were embedded in paraffin, and the H&E staining of histological sections (approximately 4 μm thick) were used to observe and evaluate the degree of hepatic fibrosis under the light microscope. The histological sectioning and its corresponding analysis were performed by an experienced pathologist. Histological findings served as the reference standard for the interpretation of the CT images of hepatic fibrosis.

### DEI Technique and Experimental Set-up

**Figure 1:**
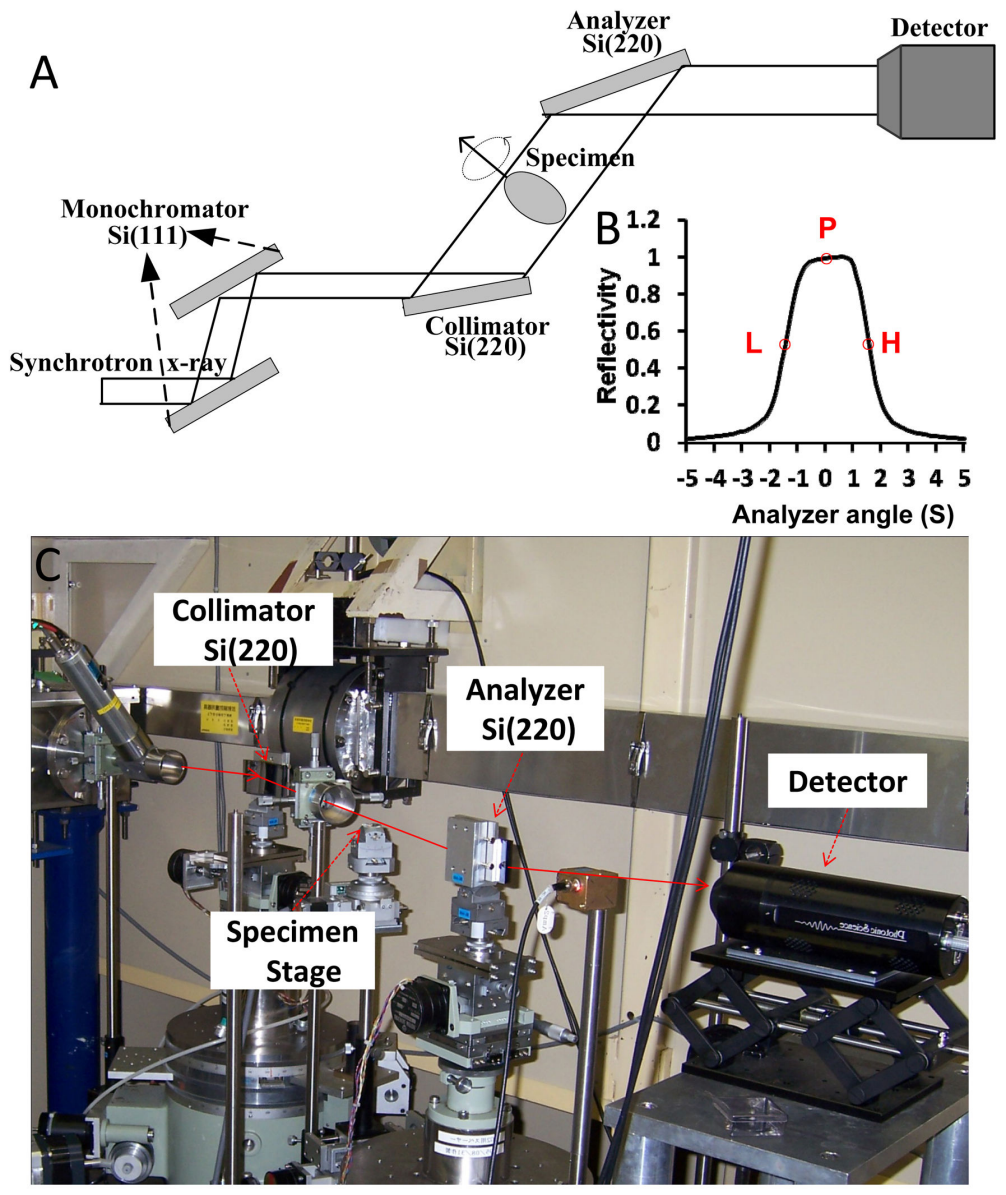
Experimental setup at beamline BL-14B in Photon Factory. (A) Schematic diagram of the DEI setup. (B) The RC of the DEI system in the experiment at an incident energy of 15 keV (1 s = 4.85 μrad). P, L, and H indicate the peak, low-angle, and high-angle positions on the RC, respectively. (C) Photograph of the optical system (top view). The double-crystal monochromator is located upstream of the experimental hutch and thus is not shown in (C).

In this study, the DEI technique was used. It is an analyzer-based x-ray imaging technique that utilizes monochromatic and collimated synchrotron radiation beams together with an analyzer crystal placed between the specimen and the detector (Figure 1A). In DEI, a monochromator crystal is used to select the energy range of the x-ray from the incident synchrotron beam and to generate a nearly monochromatic x-ray beam. Then, the beam is analyzed with a tunable analyzer crystal. The angular acceptance or reflectivity of the analyzer crystal is characterized by its rocking curve (RC). The RC describes the reflectivity of the analyzer crystal as a function of the incident angle, and it has a full width half maximum (FWHM) typically of the order of a few microradians. The analyzer crystal, which can be detuned along its RC, still acts as an angular filter for the radiation transmitted through the specimen. The crystal is able to modulate the x-ray beam by converting a small x-ray propagation angular change to a variation in the intensity that reaches the detector.

DEI image contrast depends on the width and position of the RC. By setting the analyzer at different angular positions along the RC, varying image contrasts can be obtained. Particularly meaningful positions are at the peak or slope of the RC, as shown in Figure 1B. When the analyzer is set at an angular position on the slope of the RC, an intensity modulation can be produced by x-ray deflection due to refraction. Moreover, the RC slope acts as a contrast amplifier. The steeper the slope, the greater is the image intensity variation due to refraction effects [13]. At the peak position of the RC, the analyzer crystal exactly parallels the monochromator, and the peak image can be obtained. The peak image has higher contrast than the conventional radiograph due to the presence of the extinction contrast caused by the scattering rejection.

The experiments were performed at beamline BL-14B in Photon Factory, KEK, Japan. The DEI setup consisted of a double-crystal monochromator, a DEI layout of two crystals, i.e., one collimator and the other analyzer, a specimen stage between them, and an x-ray charge coupled device (CCD) camera (Figure 1). The x-ray beam energy in the experiments was set to 15 keV. The detector employed a two-dimensional x-ray CCD camera system that had a field of view of 8.7×6.9 mm^2^. The x-ray camera was a 16-bit charge-coupled-device camera (x-ray FDI camera; Photonic Science, East Sussex, UK) with a matrix size of 1384 × 1032, a pixel size of 6.7 × 6.7 μm^2^. In the DEI imaging system, a parallel white beam emerging from the accelerator was monochromatized by a Si (111) double-crystal monochromator in a symmetrical arrangement. The monochromatic beam was incident on the asymmetrically cut Si (220) monochromator crystal with an asymmetry angle of 9.5°, which widened the beam width and reduced its angular divergence. Then, the beam transmitted through the specimen was incident on the symmetrically cut Si (220) analyzer crystal, and the intensity of the x-ray beam reflected by the analyzer crystal was measured by a detector, which resulted in the formation of DEI images. 

For DEI-CT, a total of 180 projection images of the specimen were obtained at intervals of 1.0° angular steps over 180° when the analyzer was set at the peak position of the RC (Figure 1B). A piece of 50-μm-thick polyimide polymer film (Kapton; Du Pont, Les Ulis, France) was rolled up into a tube. This type of film is an electrical insulating material with excellent thermal, mechanical and chemical properties. The specimen was contained in this tube and mounted on the rotating specimen stage, which was controlled by a precise step motor. For each projection, 10 flat-field images were recorded with no specimen in the beam. The average of these images was used to normalize the image intensity of the specimen to account for non-uniformity of the incident beam. Additionally, 10 dark images, which were recorded when no photons hit the detector, were collected, and their average was subtracted from all images on a pixel-by-pixel basis to correct for the detector’s dark current offset.

In the DEI setup, the conventional radiography experimental setup, which was based on a synchrotron source, was constructed by setting the monochromator and analyzer crystals in parallel and placing the specimen behind the analyzer crystal and close to the detector. Note that the conventional radiograph was obtained using a synchrotron light source instead of an ordinary x-ray tube. Therefore the conventional radiograph has somewhat better image quality than one would expect from a clinical radiography system [24].

### Image reconstruction and 3D visualization

During the image reconstruction and 3D visualization, the projection images were firstly performed flat-field and dark-field correction, and an image smoothing method was used to remove noise. Then, the calibration of geometric misalignment was implemented using the relative spatial position between the axis of the rotation stage and the detector in the parallel CT system. Finally, the CT images were reconstructed using a standard filter back-projection (FBP) algorithm. Image enhancement was used to highlight the vessel edges of the reconstructed CT images after ring artifact correction. The 3D vessel microstructures were visualized using 3D visualization software (Amira; Visage Imaging, Berlin, Germany) that allowed clear images of the anatomical and pathological features of the vessels.

### Image Analysis

Vessel distortion deformation and vessel tissue textures on the vessel inner wall were characterized as differences between different stages of hepatic fibrosis, and a detailed explanation is provided in later sections [2,4]. In addition, the development of fibrosis may cause compression on the vessels, and thus, the vessel branch angles obviously show stiff changes [25,26]. The vessel branch angles were calculated as a useful metric to quantify vessel pathology associated with hepatic fibrosis development. In severe hepatic fibrosis stages, a thrombus (fibrinous clot) may form in a blood vessel [27], and vessel occlusion or stenosis is usually caused by thrombi, which in many cases may lead to visible hepatic infarcts. In practice, the objective evaluation of the stenosis rate has an important value, and the stenosis rate can be evaluated based on the ratio between the thrombus area and the vessel cross-sectional area.

Because fiber hyperplasia squeezes the vessels, hepatic fibrosis can lead to vessel distortion deformation [4]. Moreover, with late-stage fibrosis, the more severe distortion deformations on large vessels become more apparent. The non-planar nature of 3D vessel tortuosity can be measured by means of torsion of its centerline. In this study, the 3D vessel centerlines were derived from the main stems of the vessels at different stages of hepatic fibrosis. Vessel torsions were calculated at a number of evenly spaced points along the length of the centerlines, and these values were used to characterize vessel distortion deformation in hepatic fibrosis. A sampling frequency of 5 points/mm was employed throughout this study, which ensured a smooth distribution of the calculated torsions.

The adhesion of platelets to collagen gives rise to a coarseness of vessel tissue textures on the vessel inner wall as fibrosis develops further [2]. The texture features, which were obtained by the gray level co-occurrence matrix (GLCM) method, were used to explore the texture changes that occurred within the vessel inner wall at different degrees of fibrosis. In this study, the texture measurements of the inverse difference moment, entropy, energy, sum average, and sum entropy were calculated using the corresponding equations. A total of 10 square regions of interest (ROIs) were derived from the vessel inner wall of the main stem at different stages of hepatic fibrosis, with each region consisting of 100×100 pixels. For the calculation of the GLCM, the original ROI image was converted to 64 gray-level image, a distance of one pixel was chosen, and four orientations (0°, 45°, 90°, and 135°) were averaged to ensure rotational invariance.

### Statistical Analyses

Statistical analyses were performed by one author with the SAS software (version 8; SAS, Cary, NC). Analysis of variance (ANOVA) was used to determine significant changes for each chosen texture parameter. All texture measurements were expressed as the means ± standard deviations. *P* values were calculated on the basis of the ANOVA, and a *P* value of less than 0.05 was considered to indicate a statistically significant difference.

## Results

### Planar X-ray Imaging

**Figure 2:**
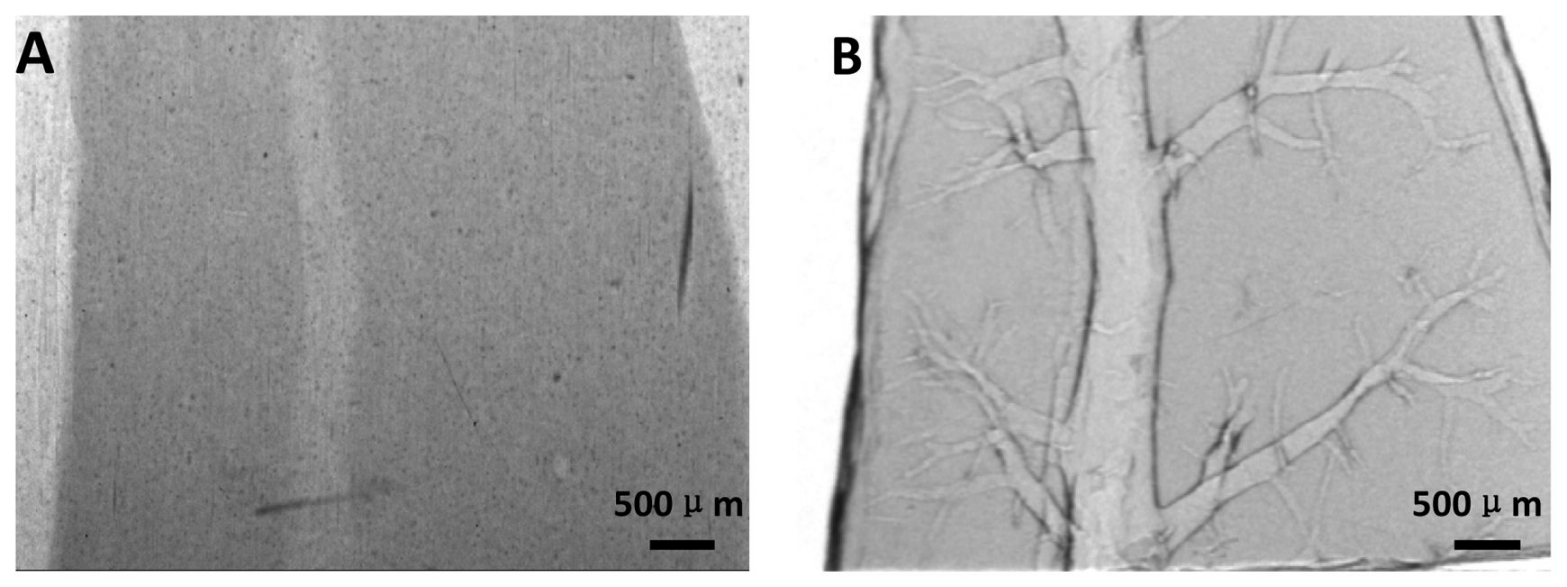
Planar x-ray images of moderate hepatic fibrosis. (A) Conventional radiograph. (B) DEI projection image.

The conventional radiograph of the liver specimen was shown in Figure 2A, and the architecture of the vessel was almost invisible. In contrast, vessel trees were clearly visualized on the micrometer scale in the DEI projection image of the same specimen (Figure 2B).

### X-ray CT Imaging and Histopathologic Analysis

**Figure 3:**
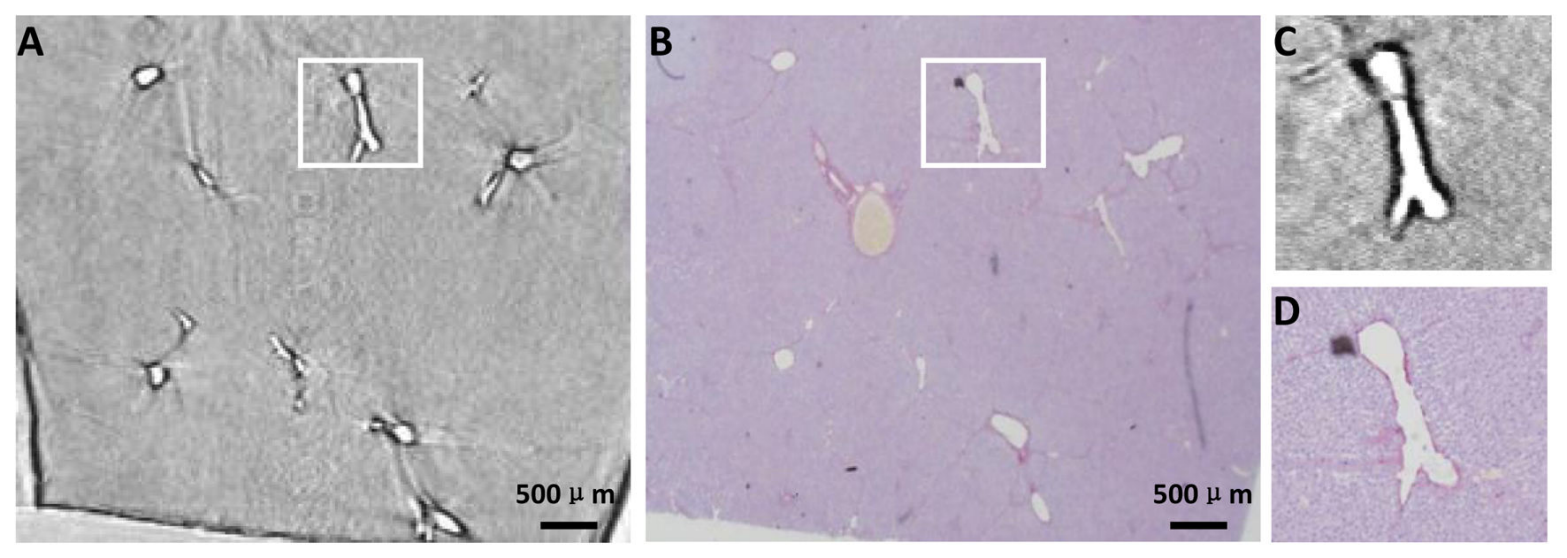
CT image and histological section of the same specimen as in Figure 2. (A) One slice of DEI-CT reconstruction images. (B) Histological section that corresponds to the area where the CT image is acquired. The two images in (C) and (D) are the enlarged images of the white rectangle regions in (A) and (B), respectively.

Figure 3A is a CT image of the specimen, and Figure 3B describes the histological section from the same specimen. The CT image had a close resemblance to the optical image of the stained histological section (Figure 3), which highlighted the high degree of sensitivity of DEI-CT.

### 3D Vessel Microstructure Visualization of Different Stages of Hepatic Fibrosis

**Figure 4:**
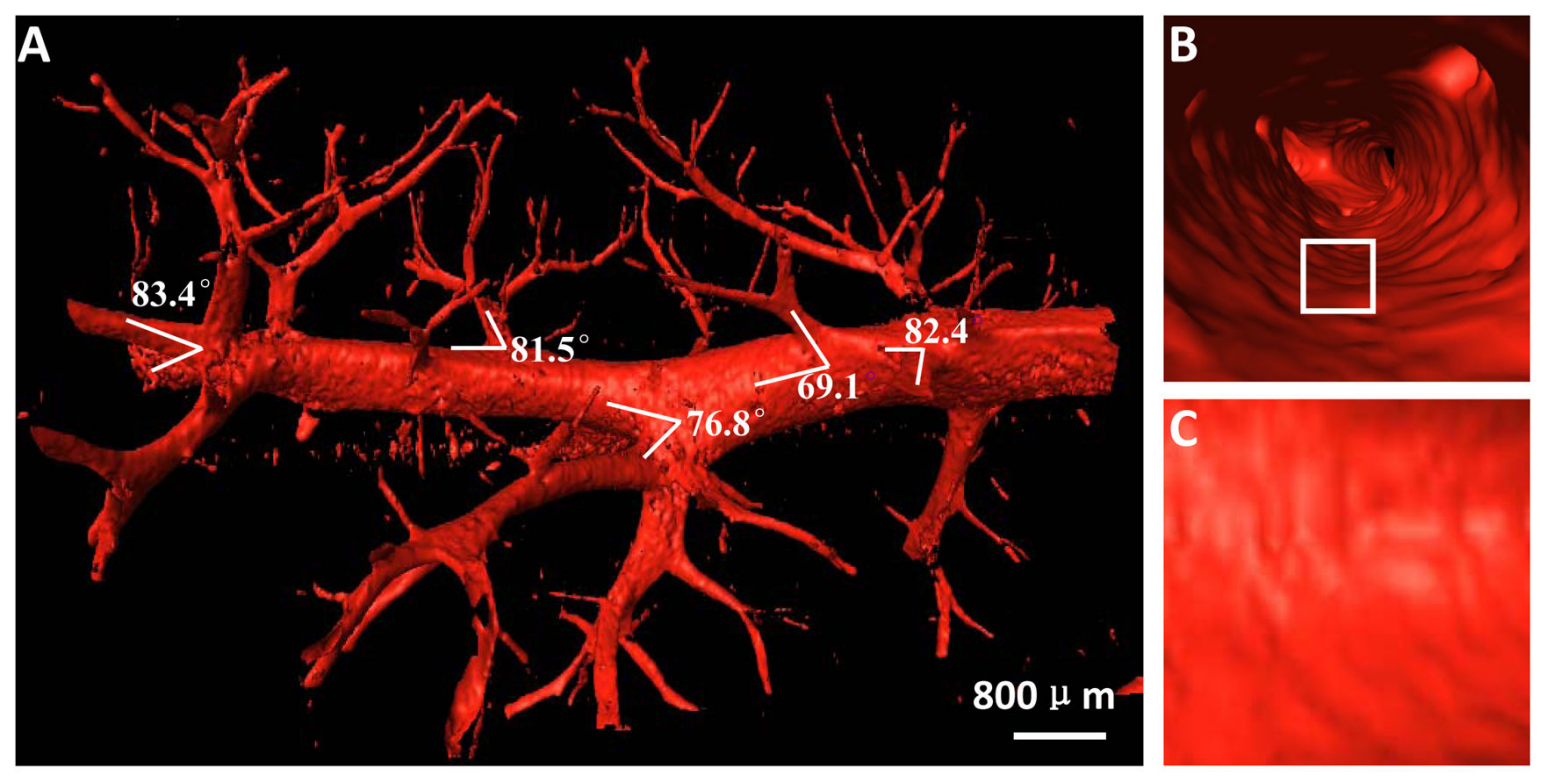
The 3D vessel microstructure image of mild hepatic fibrosis. (A) 3D vessel image. See Video S1. (B) The structure of the vessel inner wall. The virtual endoscope video is presented in the supplementary data (Video S2). (C) The enlarged image of the white rectangle region in (B).

The 3D vessel microstructure of mild hepatic fibrosis presented with vivid shapes and stereoscopic effects (Figure 4A). The structures of vessel branch, vessel trend and vessel morphology were clearly depicted, and the minimum blood vessel diameter was on the order of tens of microns. As expected, the vessel structures appeared ordered and regular without obvious vascular abnormalities, such as vascular distortion and deformation, and the vessel branches with normal angles were gradually thinned. The vessel branch angles of mild hepatic fibrosis showed less stiff changes, mostly characterizing acute angles. For better visualization of the vessel branch angle, the 3D model can be rotated in real time to facilitate an exact assessment of the branch angle (See Video S1). Figure 4B presented the structure of the vessel inner wall, and a virtual endoscope video was also provided in the supplementary data (Video S2). The vessel walls were quite smooth in mild hepatic fibrosis (Figures 4B, C).

**Figure 5:**
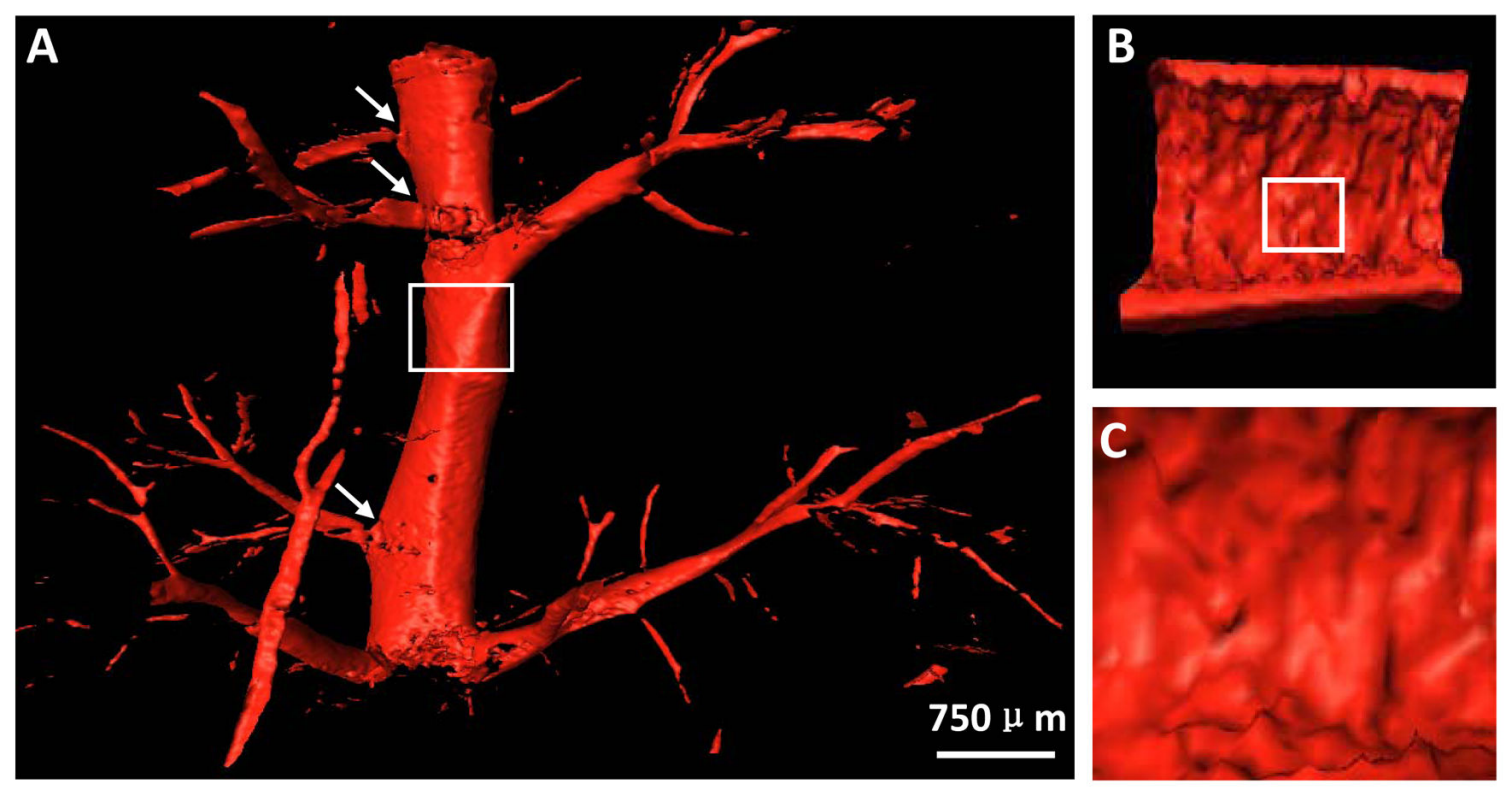
The 3D vessel microstructure image of moderate hepatic fibrosis. (A) 3D vessel image. The vessel branch angles, as indicated by the arrows, show obvious stiffness changes. (B) Longitudinal section of the vessels shown in the white rectangle region in (A). (C) The enlarged image of the white rectangle regions in (B).

Figure 5A showed the 3D vessel microstructure of moderate hepatic fibrosis. Vessel deformations, such as vessel disorders and abnormal vessel enlargement, became apparent due to the compression caused by fibrosis, and the stiffness changes, as indicated by the arrows, emerged in the vessel branch angles. Additionally, platelets adhered to the collagen surface of the injured vessel inner wall, which caused coarseness in vessel tissue textures (Figures 5B, C).

**Figure 6:**
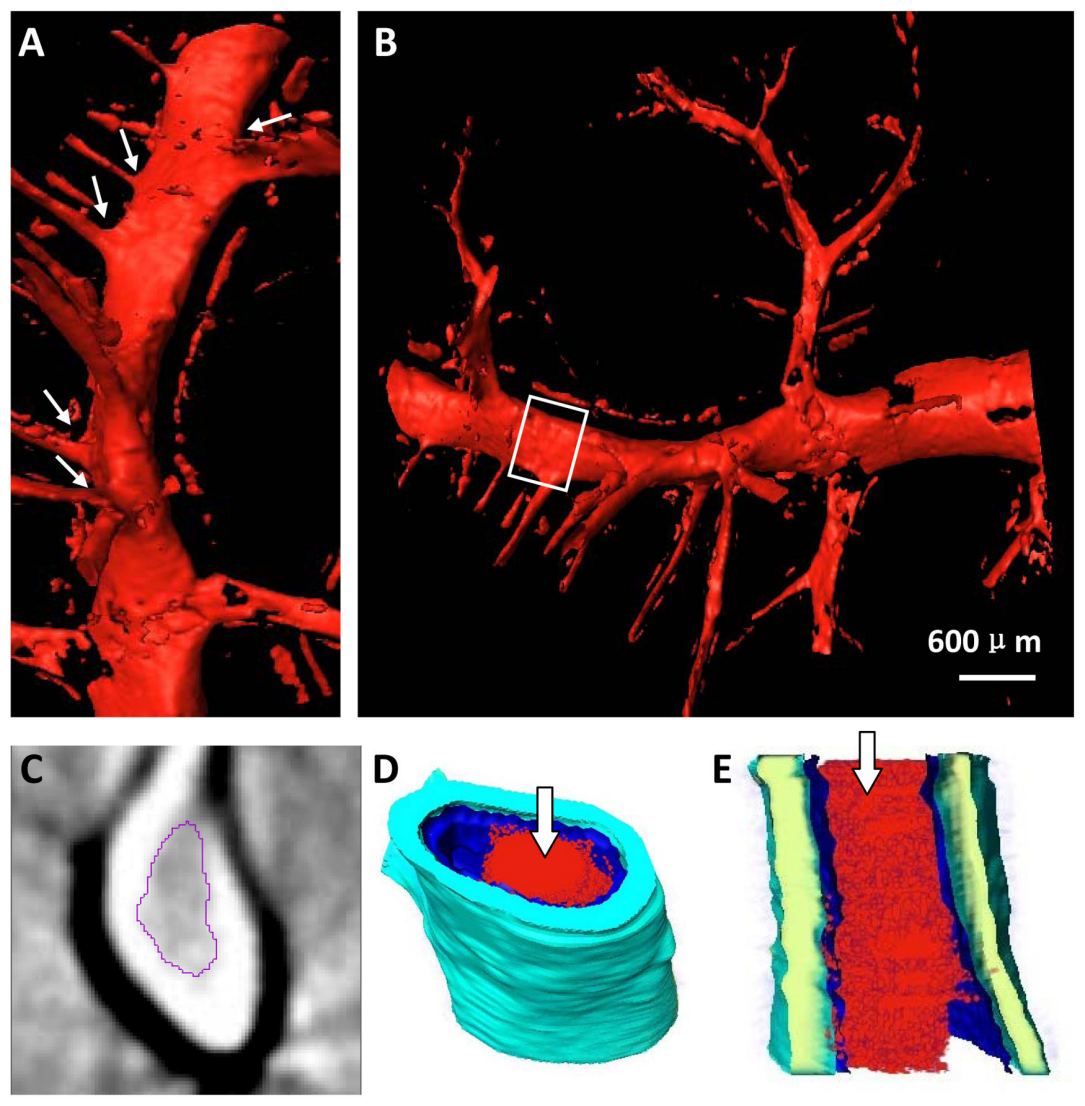
The two profiles of the 3D reconstruction are shown in (A) and (B). Obvious distortion deformation is presented in (A), and the vessel branch angles, as indicated by the arrows, appear stiffer. (C) One CT slice and (D) the vessel segment, shown in the white rectangle region in (B), are presented. The thrombus inside the vessel in (C) is segmented. (E) Longitudinal section of the vessel segment in (D). The inner red part indicated by the arrows in (D) and (E) is a thrombus.

The 3D vessel microstructure of severe hepatic fibrosis was shown in Figures 6A, B. Vessel deformations, such as decreased sub-branching, abnormal vessel trend and morphology, became more apparent as the fibrosis stage increased. The main stem of the vessel was obviously distorted, and the angles between the vessel branch and the main stem, as indicated by the arrows, appeared stiffer (Figure 6A). In advanced stages, widespread fibrosis may obscure the vessel architecture, showing an abrupt occlusion of the vessels, and the vessel mostly appeared as a ‘dry stick’ (Figure 6B). Moreover, platelets continuously adhered to the bare collagen surface of the injured vessel inner wall and were firmly immobilized on the surface of the injured vessel intima, forming an irreversible thrombosis. The thrombus inside the vessel was segmented (Figure 6C); this thrombus was invisible in conventional x-ray images. The 3D structure of the thrombus inside the chosen vessel segment was presented, which consisted of 60 DEI-CT slices, and the thrombus structure, described as the inner red part, was clearly revealed (Figures 6D, E). For the chosen vessel in Figure 6D, the quantitative measurement of the stenosis rate in different slices can be provided based on the ratio between the thrombus area and the vessel cross-sectional area.

### Vascular Distortion Deformation Analysis

**Figure 7:**
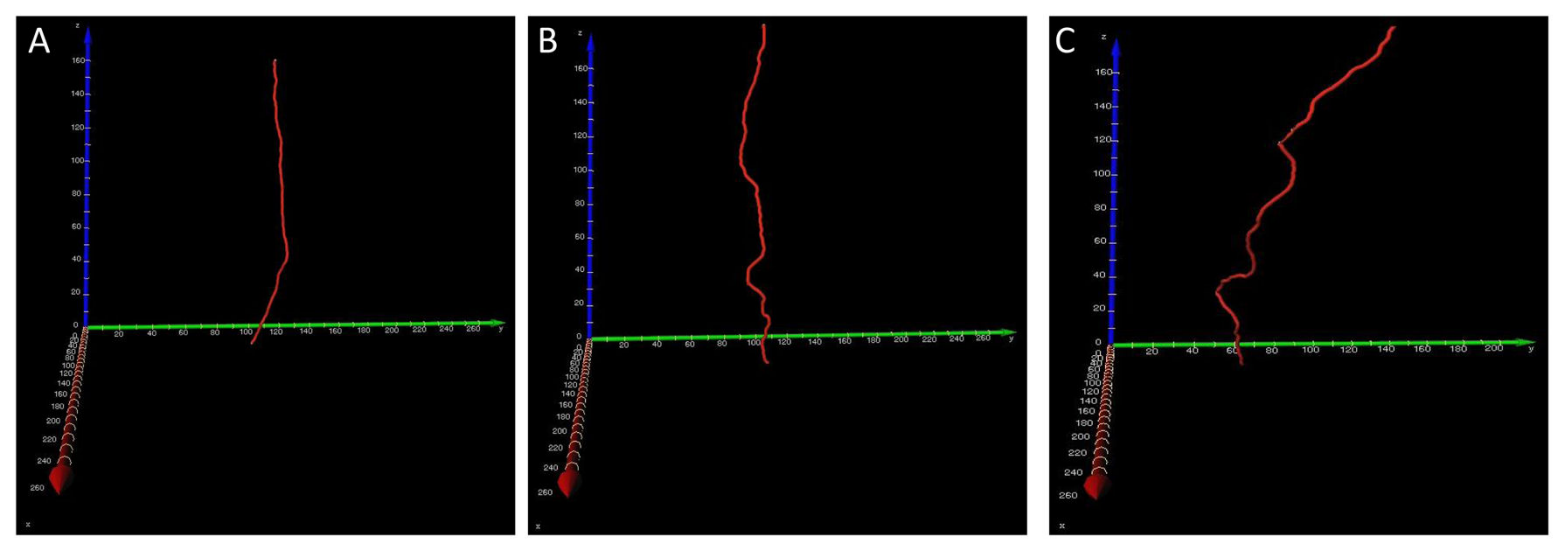
The 3D centerline images from the main stems of the vessels shown in Figures 4A, 5A and 6A. The 3D centerlines at different degrees of fibrosis can be rotated in real time to facilitate a clear visualization of the vessel distortion deformation. See Video S3, S4 and S5, respectively.

Figure 7 showed the 3D centerlines from the main stems of the vessels shown in Figures 4A, 5A and 6A, and they can be rotated in real time to facilitate a clear visualization of the vessel distortion deformation (See Videos S3, S4 and S5, respectively). The quantitative measurements of the normalized torsions at different points of the corresponding centerlines can be obtained, and the statistical analysis of torsions was presented in Table 1. With fibrosis further developed, vascular distortion deformation on the main stem of the vessels became more apparent, and the frequency of severe distortion deformations rapidly increased (Table 1).

**Table 1:** Statistical analysis of torsions.

Specimen	Mean	Standard deviation	Maximum	Minimum	Times
Mild	-0.024	0.086	0.121	-0.194	0
Moderate	-0.061	0.190	0.210	-0.731	1
Severe	0.047	0.323	0.965	-0.946	4

Note.—Times indicate statistical numbers when the absolute value of the normalized torsion is higher than 0.5.

### Quantitative Texture Analysis of the Vessel Inner Wall

The ten square ROIs, which derived from the vessel inner wall of the main stems shown in Figures 4A, 5A and 6A, respectively, were presented (See Figure S1 in supplementary material). In each ROI, the texture measurements were calculated, as summarized in Table 2. All texture features showed a statistically significant difference between different stages of fibrosis. Texture features provided quantitative descriptors that could be linked to specific fibrosis progression. Moreover, the quantitative texture analysis showed that the texture features offered objective and useful metrics to quantify vessel inner wall pathologies associated with fibrosis development.

**Table 2:** Results of the texture measurements.

Specimen	Energy	Entropy	Inverse difference moment	Sum average	Sum entropy
Mild	0.029±0.008	0.164±0.026	0.742±0.021	59.035±6.798	3.441±0.221
moderate	0.022±0.002	0.230±0.019	0.602±0.050	48.029±2.321	3.653±0.107
Severe	0.010±0.001	0.294±0.011	0.511±0.038	37.230±3.752	3.903±0.047
*P* value	<.001	<.001	<.001	<.001	<.001

Note.—Data are means ± standard deviations. Data were significantly different (*P* values of <.001 for all texture measurements).

## Discussion

Planar DEI images have been shown to reveal anatomical and pathological features associated with hepatic fibrosis without any contrast agent [4,6]. Planar imaging with DEI may not be suitable for vessels due to the overlapping of structures. DEI-CT can be used to overcome this limitation, and it can provide a clear visualization of vessel microstructures inside the specimen on the order of microns. The present study provides evidence that high-resolution DEI-CT depicts microvessel anatomical details that could otherwise only be seen using histopathology. A comparison between CT images and the histological sections of the specimens highlights the high degree of sensitivity of the DEI-CT technique. Additionally, the quality and sensitivity of the DEI-CT technique are qualitatively and quantitatively assessed by analysis of vessel morphology and structure from different stages of hepatic fibrosis. Quantitative evaluation of some vessel features, such as vascular distortion deformation and texture features on the vessel inner wall, shows a significant difference between different degrees of hepatic fibrosis and thus demonstrates the feasibility of noninvasive analysis of hepatic fibrosis. Further development of hepatic fibrosis can give rise to thrombus formation, resulting in the occlusion of the vessels. Early and accurate thrombus detection will reduce the risk of stroke and could even save lives. The DEI-CT technique presents clear visualization of the vessel internal structures, and the thrombus inside the vessel, which may not be discerned in conventional radiographs, can be accurately segmented and displayed. Quantitative analysis of the thrombus can provide exact measurements of the stenosis rate for the assessment of vessel stenosis.

The DEI-CT images in this study were acquired at the peak position of the RC. The contrast in the peak image arises mostly from the reduction of intensity owing to scattering rejection and absorption of the specimen. The presence of the extinction contrast makes the image contrast higher than the traditional x-ray image, thus resulting in high-contrast CT images. DEI derives contrast from a specimen’s x-ray absorption, refraction and ultra-small angle x-ray scattering (USAXS) properties, and these contrast mechanisms provide information about the tissue structure on different scales [24,28]. In fact, the refraction and USAXS contrast mechanisms are ideally suited for soft tissues imaging because of the improved sensitivity, and they can potentially reveal valuable information regarding tissue microstructures. For the successful implementation of refraction and USAXS contrast, DEI-CT images should be acquired at many points along the RC, and several algorithms have so far been proposed [29,30]. Based on the refraction and USAXS contrast mechanisms, further investigation of hepatic fibrosis with DEI-CT images is in progress. Additionally, in CT imaging for soft tissues, the radiation dose delivered to the specimen is an important research issue, and dose minimization is a fundamental goal for researchers. In recent years, the compressed sensing (CS)-based iterative algorithm and equally sloped tomography (EST) algorithm have been incorporated into the DEI-CT reconstruction, and the corresponding algorithms can accurately reconstruct CT images using substantially reduced radiation dose compared to the traditional FBP algorithm [31,32]. This development provides great promise for the use of CT imaging in biomedical applications.

It is evident that DEI-CT technique provides improved detail visibility of the vessel morphology features, and there is a correlation between the degree of hepatic fibrosis and vessel features. Further investigations should be explored because the preliminary results of this study are encouraging. In practice, valuable information, including the structures of hepatic lobules, pseudolobules and fiber septa, can play a crucial role in the evaluation of the degree of hepatic fibrosis and progression. This information can be obtained using DEI-CT technique, which will be very helpful to better understand pathology correlations. In the present feasibility study, the number of samples is very small. More specimens of different stages of hepatic fibrosis need to be studied to achieve statistically significant differences in DEI-CT images.

In past years, DEI-CT has opened new avenues for biological soft tissues imaging, owing to the unprecedented spatial and contrast resolution, and its applications have covered a wide range of pathologies and organs [33]. Recently, a large field of view DEI-CT system has been developed, producing high-contrast images of large and complex specimens [18,32,34]. Moreover, it has been shown that DEI-CT still can maintain high sensitivity at high x-ray energies, which opens the way to dose reduction [18,32,35]. Compared with high x-ray energies, DEI-CT can provide the high-resolution 3D information of soft tissues and tumors in whole and large breasts, and deliver less radiation doses to the sample [18,32]. Currently, the DEI images are mostly acquired using synchrotron radiation, which hinders its clinical application. Fortunately, a large field of view DEI system using an x-ray tube source is under development, and several novel techniques have been demonstrated [36-38]. This will be an important step in the evolution of DEI-CT towards its clinical implementation. The isolated animal specimens were the main subjects of our experiments. To evaluate the DEI-CT value in hepatic fibrosis diagnosis, it would be very important to determine if clinically relevant diagnostic information can be obtained in vivo with this technique based on human tissues. In principle, the high-resolution and low-dose DEI-CT images can be acquired in vivo. Proof-of-principle studies of in vivo application of DEI-CT have been performed to investigate the development of osteoarthritis [39]. Additionally, dual-energy CT allows reconstruction of virtual monochromatic images avoiding the use of synchrotron radiation [40]. A new technique that combines the idea of dual energy with PCI is presented, and it can provide high sensitivity for weakly absorbing materials such as polymers and soft tissues [41]. These researches demonstrate the possibility of implementation of the DEI-CT in a clinical environment, which will provide access to a practical, clinical diagnosis for hepatic fibrosis. Thus, with further development and validation of the technology, the noninvasive diagnosis of hepatic fibrosis with DEI-CT may become routine clinical practice.

## Supporting Information

Figure S1 **The regions of interest from the vessel inner wall of the main stem at different degrees fibrosis.** These regions originate from arbitrary selections in corresponding vessel inner walls.
(TIF)

Video S1 **Animated view of the 3D vessel microstructure image of a mild hepatic fibrosis specimen.** This is the same 3D model shown in Figure 4A. The rotation of the model permits the viewers to better observe the vessel branch angle, and facilitate an exact assessment of the branch angle.
(AVI)

Video S2 **The virtual endoscope inside the vessels.** This is the same model shown in Figure 4B. The virtual endoscope of the model is provided to clearly present the structure of the vessel inner wall.
(AVI)

Video S3 **Animated view of the 3D centerline from the main stem of the vessels shown in Figure 4A.** This is the same 3D model shown in Figure 7A.
(AVI)

Video S4 **Animated view of the 3D centerline from the main stem of the vessels shown in Figure 5A.** This is the same 3D model shown in Figure 7B.
(AVI)

Video S5 **Animated view of the 3D centerline from the main stem of the vessels shown in Figure 6A.** This is the same 3D model shown in Figure 7C.
(AVI)

## References

[1] Schuppan D, Afdhal NH (2008) Liver cirrhosis. Lancet 371: 838-851. doi:10.1016/S0140-6736(08)60383-9. PubMed: 18328931. 18328931 PMC2271178

[2] Bataller R, Brenner DA (2005) Liver fibrosis. J Clin Invest 115: 209-218. doi:10.1172/JCI200524282. PubMed: 15690074. 15690074 PMC546435

[3] Patel KD, Abeysekera KWM, Marlais M, McPhail MJ, Thomas HC et al. ( 2011) Recent advances in imaging hepatic fibrosis and steatosis. Expert. Rev Gastroenterol Hepatol 5: 91-104. doi:10.1586/egh.10.85. 21309675

[4] Li H, Zhang L, Wang XY, Wang TL, Wang BE et al. (2009) Investigation of hepatic fibrosis in rats with x-ray diffraction enhanced *i*maging. Appl Phys Lett 94: 124101 (3 pp.) doi:10.1063/1.3104860.

[5] Zhang L, Hu CH, Zhao T, Luo SQ (2011) Noninvasive visualization of microvessels using diffraction enhanced imaging. Eur J Radiol 80: 158-162. doi:10.1016/j.ejrad.2010.08.019. PubMed: 20833491. 20833491

[6] Zhang X, Yang XR, Chen Y, Li HQ, Li RM et al. (2013) Visualising liver fibrosis by phase-contrast X-ray imaging in common bile duct ligated mice. Eur Radiol 23: 417-423. doi:10.1007/s00330-012-2630-z. PubMed: 22903640. 22903640

[7] Bonekamp S, Kamel I, Solga S, Clark J (2009) Can imaging modalities diagnose and stage hepatic fibrosis and cirrhosis accurately. J Hepatol 50: 17-35. doi:10.1016/j.jhep.2008.10.016. PubMed: 19022517. 19022517

[8] Godfrey EM, Mannelli L, Griffin N, Lomas DJ (2013) Magnetic resonance elastography in the diagnosis of hepatic fibrosis. Semin Ultrasound CT MR 34: 81-88. doi:10.1053/j.sult.2012.11.007. PubMed: 23395320. 23395320

[9] Caro JJ, Trindade E, McGregor M (1991) The risks of death and of severe nonfatal reactions with high- vs low-osmolality contrast media: a meta-analysis. AJR Am J Roentgenol 156: 825-832. doi:10.2214/ajr.156.4.1825900. PubMed: 1825900. 1825900

[10] Momose A, Takeda T, Itai Y, Hirano K (1996) Phase-contrast x-ray computed tomography for observing biological soft tissues. Nat Med 2: 473-475. doi:10.1038/nm0496-473. PubMed: 8597962. 8597962

[11] Davis TJ, Gao D, Gureyev TE, Stevenson AW, Wilkins SW (1995) Phase-contrast imaging of weakly absorbing materials using hard x-rays. Nature 373: 595-598. doi:10.1038/373595a0.

[12] Wilkins SW, Gureyev TE, Gao D, Pogany A, Stevenson AW (1996) Phase-contrast imaging using polychromatic hard X-rays. Nature 384: 335-338. doi:10.1038/384335a0.

[13] Chapman D, Thomlinson W, Johnston RE, Washburn D, Pisano E et al. (1997) Diffraction enhanced x-ray imaging. Phys Med Biol 42: 2015-2025. doi:10.1088/0031-9155/42/11/001. PubMed: 9394394. 9394394

[14] Pisano ED, Johnston RE, Chapman D, Geradts J, Iacocca MV et al. (2000) Human breast cancer specimens: Diffraction-enhanced imaging with histologic correlation-improved conspicuity of lesion detail compared with digital radiography. Radiology 214: 895-901. PubMed: 10715065. 10715065 10.1148/radiology.214.3.r00mr26895

[15] Arfelli F, Bonvicini V, Bravin A, Cantatore G, Castelli E et al. (2000) Mammography with synchrotron radiation: phase-detection techniques. Radiology 215: 286-293. PubMed: 10751500. 10751500 10.1148/radiology.215.1.r00ap10286

[16] Bravin A, Keyriläinen J, Fernández M, Fiedler S, Nemoz C et al. (2007) High-resolution CT by diffraction-enhanced x-ray imaging: mapping of breast tissue samples and comparison with their histo-pathology. Phys Med Biol 52: 2197-2211. doi:10.1088/0031-9155/52/8/011. PubMed: 17404464. 17404464

[17] Keyriläinen J, Fernández M, Karjalainen-Lindsberg ML, Virkkunen P, Leidenius M et al. (2008) Toward high-contrast breast CT at low radiation dose. Radiology 249: 321-327. doi:10.1148/radiol.2491072129. PubMed: 18796684. 18796684

[18] Sztrókay A, Diemoz PC, Schlossbauer T, Brun E, Bamberg F et al. (2012) High-resolution breast tomography at high energy: a feasibility study of phase contrast imaging on a whole breast. Phys Med Biol 57: 2931-2942. doi:10.1088/0031-9155/57/10/2931. PubMed: 22516937. 22516937

[19] Connor DM, Benveniste H, Dilmanian FA, Kritzer MF, Miller LM et al. (2009) Computed tomography of amyloid plaques in a mouse model of Alzheimer's disease using diffraction enhanced imaging. Neuroimage 46: 908-914. doi:10.1016/j.neuroimage.2009.03.019. PubMed: 19303447. 19303447 PMC4652842

[20] Seo SJ, Sunaguchi N, Yuasa T, Huo QK, Ando M et al. (2012) Visualization of microvascular proliferation as a tumor infiltration structure in rat glioma specimens using the diffraction-enhanced imaging in-plane CT technique. Phys Med Biol 57: 1251-1262. doi:10.1088/0031-9155/57/5/1251. PubMed: 22330695. 22330695

[21] Hu CH, Zhao T, Zhang L, Li H, Zhao XY et al. (2009) Information extraction and CT reconstruction of liver image based on diffraction enhanced imaging. Prog Nat Sci 19: 955-962. doi:10.1016/j.pnsc.2008.06.031.

[22] Zhao XY, Zeng X, Li XM, Wang TL, Wang BE (2009) Pirfenidone inhibits carbon tetrachloride- and albumin complex-induced liver fibrosis in rodents by preventing activation of hepatic stellate cells. Clin Exp. Pharmacologist: 36: 963-968 10.1111/j.1440-1681.2009.05194.x 19413596

[23] Zhao XY, Wang BE, Li XM, Wang TL (2008) Newly proposed fibrosis staging criterion for assessing carbon tetrachloride- and albumin complex-induced liver fibrosis in rodents. Clin. Pathol Int 58: 580-588. doi:10.1111/j.1440-1827.2008.02274.x. PubMed: 18801073. 18801073

[24] Wernick MN, Wirjadi O, Chapman D, Zhong Z, Galatsanos NP et al. (2003) Multiple-image radiography. Phys Med Biol 48: 3875-3895. doi:10.1088/0031-9155/48/23/006. PubMed: 14703164. 14703164

[25] Lu JS, Di RK, Wu YM, Tang XX, Miao DS (1994) Morphological observation on the portal vein and its tributaries in children. Chinese journal of anatomy 17: 220-223

[26] Liang KH, Li SB (1999) Portal hypertension. Beijing: People's Military. Med Press. 7p.

[27] Papatheodoridis GV, Papakonstantinou E, Andrioti E Cholongitas E, Petraki K et al. (2003) Thrombotic risk factors and extent of liver fibrosis in chronic viral hepatitis. Gut 52: 404-409. 12584224 10.1136/gut.52.3.404 PMC1773539

[28] Muehleman C, Li J, Zhong Z, Brankov JG, Wernick MN (2006) Multiple-image radiography for human soft tissue. J Anat 208: 115-124. doi:10.1111/j.1469-7580.2006.00502.x. PubMed: 16420384. 16420384 PMC2100186

[29] Brankov JG, Wernick MN, Yang YY, Li J, Muehleman C et al. (2006) Computed tomography implementation of multiple-image radiography. Med Phys 33: 278-289. doi:10.1118/1.2150788. PubMed: 16532932. 16532932

[30] Diemoz PC, Bravin A, Glaser C, Coan P (2010) Comparison of analyzer-based imaging computed tomography extraction algorithms and application to bone-cartilage imaging. Phys Med Biol 55: 7663-7679. doi:10.1088/0031-9155/55/24/018. PubMed: 21113091. 21113091

[31] Li XL, Luo SQ (2011) A compressed sensing-based iterative algorithm for CT reconstruction and its possible application to phase contrast imaging. Biomed Eng Available: 10: 773 (14 pp.) 10.1186/1475-925X-10-73 PMC3169509 21849088

[32] Zhao YZ, Brun E, Coan P, Huang ZF, Sztrókay A et al. (2012) High-resolution, low-dose phase contrast X-ray tomography for 3D diagnosis of human breast cancers. Proc Natl Acad Sci U_S_A 109: 18290-18294. doi:10.1073/pnas.1204460109. PubMed: 23091003. 23091003 PMC3494902

[33] Bravin A, Coan P, Suortti P (2013) X-ray phase-contrast imaging: from pre-clinical applications towards clinics. Phys Med Biol 58: R1-R35. doi:10.1088/0031-9155/58/1/1. PubMed: 23220766. 23220766

[34] Gasilov S, Mittone A, Brun E, Bravin A, Grandl S et al. (2013) On the possibility of quantitative refractive-index tomography of large biomedical samples with hard X-rays Biomed Opt Express 4: 1512-1518. 10.1364/BOE.4.001512 PMC3771823 24049673

[35] Keyriläinen J, Fernández M, Bravin A, Karjalainen-Lindsberg ML, Leidenius M et al. (2011) Comparison of in vitro breast cancer visibility in analyser-based computed tomography with histopathology, mammography, computed tomography and magnetic resonance imaging. J Synchrotron Radiat 18: 689-696. doi:10.1107/S090904951102810X. PubMed: 21862846. 21862846

[36] Faulconer L, Parham C, Connor DM, Zhong Z, Kim E et al. (2009) Radiologist evaluation of an x-ray tube-based diffraction-enhanced imaging prototype using full-thickness breast specimens. Acad Radiol 16: 1329-1337. doi:10.1016/j.acra.2009.05.006. PubMed: 19596593. 19596593

[37] Muehleman C, Fogarty D, Reinhart B, Tzvetkov T, Li J et al. (2010) In-laboratory diffraction-enhanced x-ray imaging for articular cartilage. Clin Anat 23: 530-538. doi:10.1002/ca.20993. PubMed: 20544949. 20544949

[38] Parham C, Zhong Z, Connor DM, Chapman LD, Pisano ED (2009) Design and implementation of a compact low-dose diffraction enhanced medical imaging system. Acad Radiol 16: 911-917. doi:10.1016/j.acra.2009.02.007. PubMed: 19375952. 19375952

[39] Coan P, Wagner A, Bravin A, Diemoz PC, Keyriläinen J et al. (2010) In vivo x-ray phase contrast analyzer-based imaging for longitudinal osteoarthritis studies in guinea pigs. Phys Med Biol 55: 7649-7662. doi:10.1088/0031-9155/55/24/017. PubMed: 21113092. 21113092

[40] Mannelli L, Mitsumori LM, Ferguson M, Xu DX, Chu BC et al. (2013) Changes in measured size of atherosclerotic plaque calcifications in dual-energy CT of ex vivo carotid endarterectomy specimens: effect of monochromatic keV image reconstructions. Eur Radiol 23: 367-374. doi:10.1007/s00330-012-2623-y. PubMed: 22907636. 22907636

[41] Kottler C, Revol V, Kaufmann R, Urban C (2010) Dual energy phase contrast x-ray imaging with Talbot-Lau interferometer. J Appl Phys 108: 114906 (6 pp.) doi:10.1063/1.3512871.

