# Supplementary figures and images for: Microcomputed Tomography with Diffraction-Enhanced Imaging for Morphologic Characterization and Quantitative Evaluation of Microvessel of Hepatic Fibrosis in Rats
Source: PLoS One. 2013 Oct 21;8(10):e78176. doi: 10.1371/journal.pone.0078176 (PMC3804625; doi:10.1371/journal.pone.0078176)

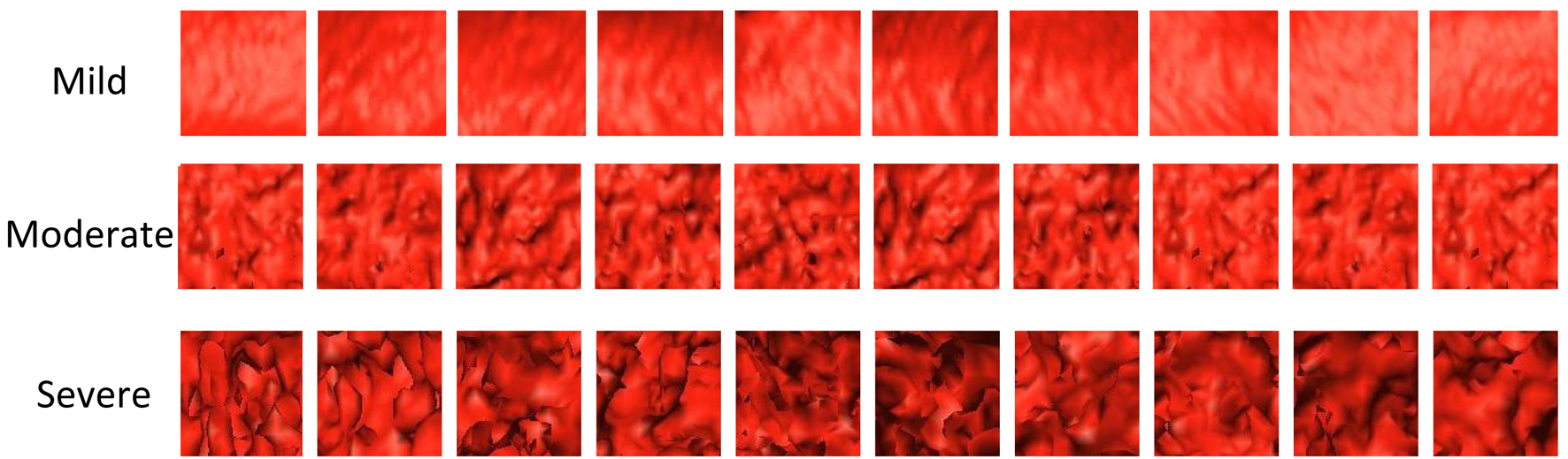

Supplement: Figure S1 — The regions of interest from the vessel inner wall of the main stem at different degrees fibrosis. These regions originate from arbitrary selections in corresponding vessel inner walls. (TIF) [file pone.0078176.s001.tif]
